# Case Report: Delayed coil migration after endovascular embolization of an inferior mesenteric arteriovenous malformation

**DOI:** 10.3389/fmed.2026.1891864

**Published:** 2026-09-18

**Authors:** Lijia Wei, Yiyuan Li, Bin Huang, Xiyang Chen

**Affiliations:** Division of Vascular Surgery, Department of General Surgery, West China Hospital, Sichuan University, Chengdu, Sichuan, China

**Keywords:** arteriovenous malformation, coil-related bowel injury, endovascular embolization, mesenteric arteriovenous lesion, open surgery

## Abstract

**Background:**

Inferior mesenteric arteriovenous malformation (IMAVM) is an exceptionally rare vascular lesion. Endovascular embolization is frequently selected as first-line therapy; however, long-term outcomes and delayed complications remain poorly defined.

**Case summary:**

A 43-year-old woman presented with a 1-year history of progressive fatigue and a 6-month history of intermittent lower abdominal pain and hematochezia, with worsening bright red rectal bleeding over the preceding 3 days. Ten years earlier, she had undergone endovascular embolization for an abdominal vascular malformation at another hospital. Computed tomography angiography (CTA) demonstrated extensive serpiginous abdominopelvic vessels, marked venous dilatation and suspected metallic material within the intestinal lumen. Colonoscopy subsequently revealed a circumferential sigmoid ulcer with luminal stenosis, fistulous openings, and intraluminal exposure of metallic embolization material. Digital subtraction angiography (DSA) demonstrated an extensive high-flow inferior mesenteric arteriovenous malformation (IMAVM), characterized by a diffuse nidus of numerous tortuous abnormal vessels involving the sigmoid mesocolon and adjacent pelvic and retroperitoneal regions. The lesion was predominantly supplied by branches of the inferior mesenteric artery, with early venous drainage through the markedly dilated left ovarian and renal veins into the inferior vena cava. Persistent opacification and recanalization of vessels previously treated by coil embolization were also observed. Ongoing hemorrhage and progressive anemia necessitated emergency hybrid surgery, including resection of the AVM nidus, left renal vein angioplasty, involved bowel segments, excision of the left ovarian venous aneurysm, Hartmann's procedure, and small-bowel anastomosis. The patient recovered uneventfully and remained asymptomatic at the two-year follow-up.

**Conclusion:**

Delayed coil migration into the gastrointestinal tract is a rare but potentially life-threatening complication of endovascular embolization for IMAVM, warranting long-term clinical and imaging surveillance. In patients with active bleeding, fistula formation, and extensive vascular or bowel involvement, surgery offers a direct and definitive approach to removing exposed embolic material and treating the associated intestinal and vascular lesions.

## Introduction

Arteriovenous fistulas involving the splanchnic circulation are uncommon vascular anomalies characterized by direct high-flow shunting between arteries and veins. According to the International Society for the Study of Vascular Anomalies (ISSVA) classification, unlike an arteriovenous fistula, an arteriovenous malformation (AVM) comprises a more complex network of abnormal arterial and venous channels (1). Among them, inferior mesenteric arteriovenous malformation (IMAVM) is an exceptionally rare high-flow vascular lesion of the splanchnic circulation, and published experience is limited largely to case reports and literature reviews (2, 3). Endovascular embolization is widely used because it can occlude the abnormal shunt while being less invasive than open surgery (4, 5). However, embolization material may occasionally erode into or migrate toward adjacent hollow viscera, resulting in ulceration, fistula formation, gastrointestinal bleeding, obstruction, or infection (6–10). Such delayed coil-related bowel complications are uncommon, and their clinical course, mechanisms, and optimal management remain poorly defined. According to the available literature, coil-related complications may develop from several days to many years after endovascular embolization (6–8). Reported cases of gastrointestinal coil migration or erosion have predominantly involved the stomach or duodenum following embolization of the gastroduodenal, hepatic, or splenic arteries, whereas colonic involvement has been reported far less frequently (6–10). Clinical manifestations include abdominal pain, gastrointestinal bleeding, perforation or fistula formation, intestinal obstruction, and secondary infection, with fatal hemorrhage occurring in rare cases (6–10). We report a patient with previously diagnosed IMAVM who developed a rare delayed complication after embolization. The embolization coil eroded into the colon, causing a circumferential sigmoid ulcer, enterocolic fistulas, and massive hematochezia in the setting of an extensive high-flow abdominopelvic vascular lesion. This case underscores the diagnostic and therapeutic challenges of delayed coil-related complications and the value of emergency hybrid surgery when active bleeding, severe bowel injury, and complex vascular involvement coexist.

## Case description

A 43-year-old female was admitted with progressive fatigue for one year and a 6-month history of abdominal pain accompanied by hematochezia, with an increase in fresh-blood hematochezia during the preceding three days. One year before admission, she developed fatigue without an apparent precipitating factor; the symptom improved with rest, and she did not seek medical treatment. Six months before admission, she began experiencing intermittent cramping or dull abdominal pain without an identifiable cause. The pain was unrelated to food intake, lasted several minutes, and resolved spontaneously. She also occasionally passed bright red bloody stools, occurring once or twice weekly, with an estimated volume of approximately 50 g per episode. She self-medicated with omeprazole and a cyclooxygenase-2 inhibitor, which partially relieved her symptoms, but she did not undergo further medical evaluation or treatment. She denied fever, weight loss, diarrhea, nausea, vomiting, melena, difficulty with defecation, or any recent change in bowel habits. Ten years earlier, she had undergone endovascular embolization for an abdominal vascular malformation at another hospital. According to the patient, she experienced no significant symptoms after the previous intervention. Despite extensive efforts, records of the previous procedure could not be obtained, and the patient had not attended the recommended postoperative follow-up. She denied any misuse of medications or herbal remedies and had no other relevant family history, psychosocial history, or known history of genetic disorders. Apart from the endovascular intervention performed 10 years earlier, she denied any history of trauma or other percutaneous interventions. Her heart rate was 88 beats/min, respiratory rate was 20 breaths/min, and blood pressure was 118/78 mmHg. Physical examination revealed mild abdominal distension without dilated abdominal wall veins. Mild tenderness was present in the left lower quadrant, without rebound tenderness. A pulsatile mass with a palpable thrill was noted in the left abdomen. No peripheral edema was observed in either lower extremity. The patient's hemoglobin level was 90 g/L, red blood cell count was 4.11 × 10^12^/L, and white blood cell count was 11.65 × 10^9^/L. Laboratory tests showed an alpha-fetoprotein level of 1.3 ng/mL, a carbohydrate antigen 19-9 level of 10.90 U/mL, a procalcitonin level of 1.220 ng/mL, and an interleukin-6 level exceeding 5,000 pg/mL. Computed tomography angiography revealed an inferior mesenteric arteriovenous malformation (Figure 1A), marked aneurysmal dilatation of the left renal vein (2.7 cm, Figure 1B) and ovarian vein (4.9 cm × 5.4 cm, Figure 1C), extensive serpiginous collateral vessels throughout the pelvis and retroperitoneum, and suspected metallic material within the bowel lumen. As the patient was hemodynamically stable, colonoscopy and immunologic testing were planned to identify the source of bleeding, while supportive medical treatment was initiated.

**Figure 1:**
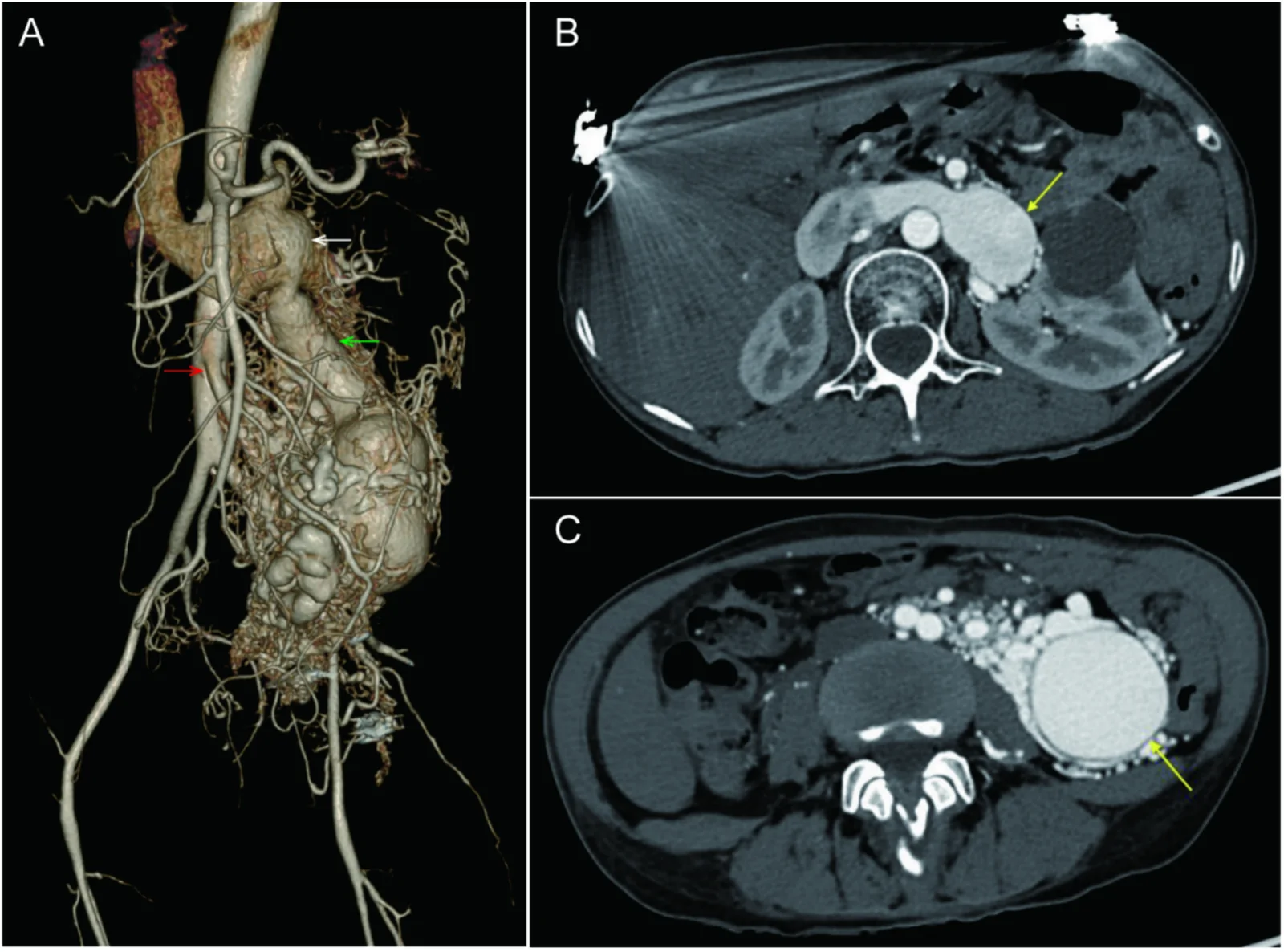
Preoperative computed tomography angiography findings. **(A)** Three-dimensional volume-rendered reconstruction demonstrates an inferior mesenteric arteriovenous malformation (red arrow), with marked dilatation of the left renal vein (white arrow) and left ovarian vein (green arrow) and venous drainage into the inferior vena cava. **(B)** Axial image shows aneurysmal dilatation of the left renal vein, measuring 2.7 cm (yellow arrow). **(C)** Axial image shows a large left ovarian venous aneurysm measuring 4.9 cm × 5.4 cm (yellow arrow), surrounded by numerous dilated collateral vessels.

Unexpectedly, on the second hospital day, the patient developed two episodes of massive hematochezia (approximately 200 g each), accompanied by hypotension (98/56 mmHg), a pulse rate of 108 beats/min, and tachypnea (23 breaths/min). An urgent complete blood count showed that the hemoglobin level had decreased to 78 g/L. Emergency colonoscopy was subsequently performed to identify the bleeding source. A circumferential ulcer with luminal narrowing was identified approximately 18 cm from the anal verge, with exposed metallic embolic material visible at its base (Figure 2A). Manipulation with biopsy forceps provoked brisk bleeding, confirming active bleeding from the ulcerated segment with coil exposure. Two deep fistulous openings were also identified in the sigmoid colon (Figure 2B). Because endoscopic manipulation precipitated bleeding and endoscopic therapy alone could not address the extensive serpiginous vascular lesions, no further attempt was made to retrieve the coil or close the fistulas endoscopically. Based on the clinical presentation and imaging findings, the diagnoses were as follows: (1) lower gastrointestinal bleeding secondary to an inferior mesenteric arteriovenous malformation, with a suspected enteric fistula; (2) compensated hemorrhagic shock; (3) coil migration following coil embolization of the inferior mesenteric arteriovenous malformation. Differential diagnoses included colorectal malignancy, inflammatory or ischemic colitis, and a primary enterocolic fistula. Colonoscopy demonstrated the exposed embolization coil and DSA revealed an extensive vascular malformation. These findings supported the final diagnosis. Given the ongoing bleeding and extensive vascular involvement, close monitoring and definitive surgery were required to achieve durable hemorrhage control. The patient's clinical timeline is shown in Figure 3.

**Figure 2:**
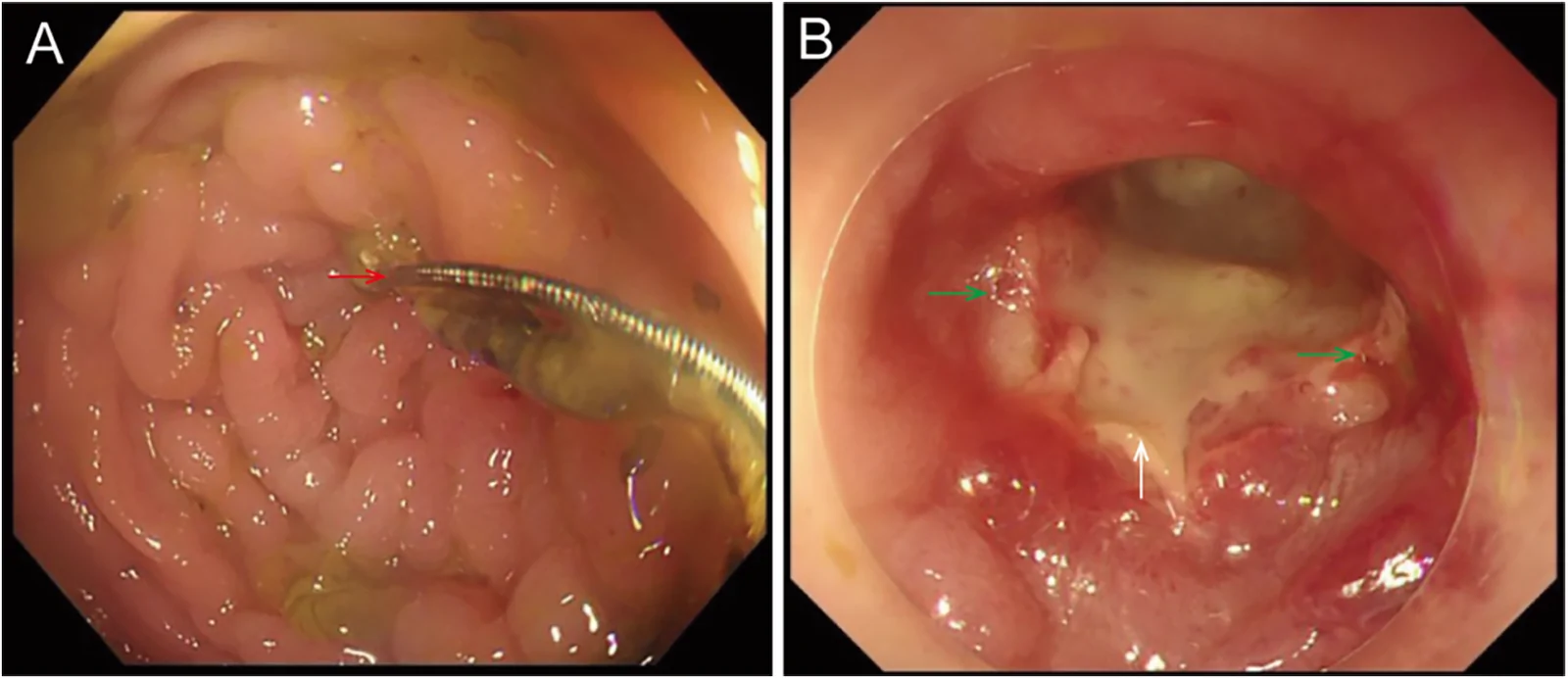
Colonoscopic findings. **(A)** Exposed metallic embolization coil (red arrow) protruding into the sigmoid colon lumen. **(B)** Two deep fistulous openings within the circumferential ulcerated segment of the sigmoid colon (green arrow), with surrounding mucosal inflammation and exudation (white arrow).

**Figure 3:**
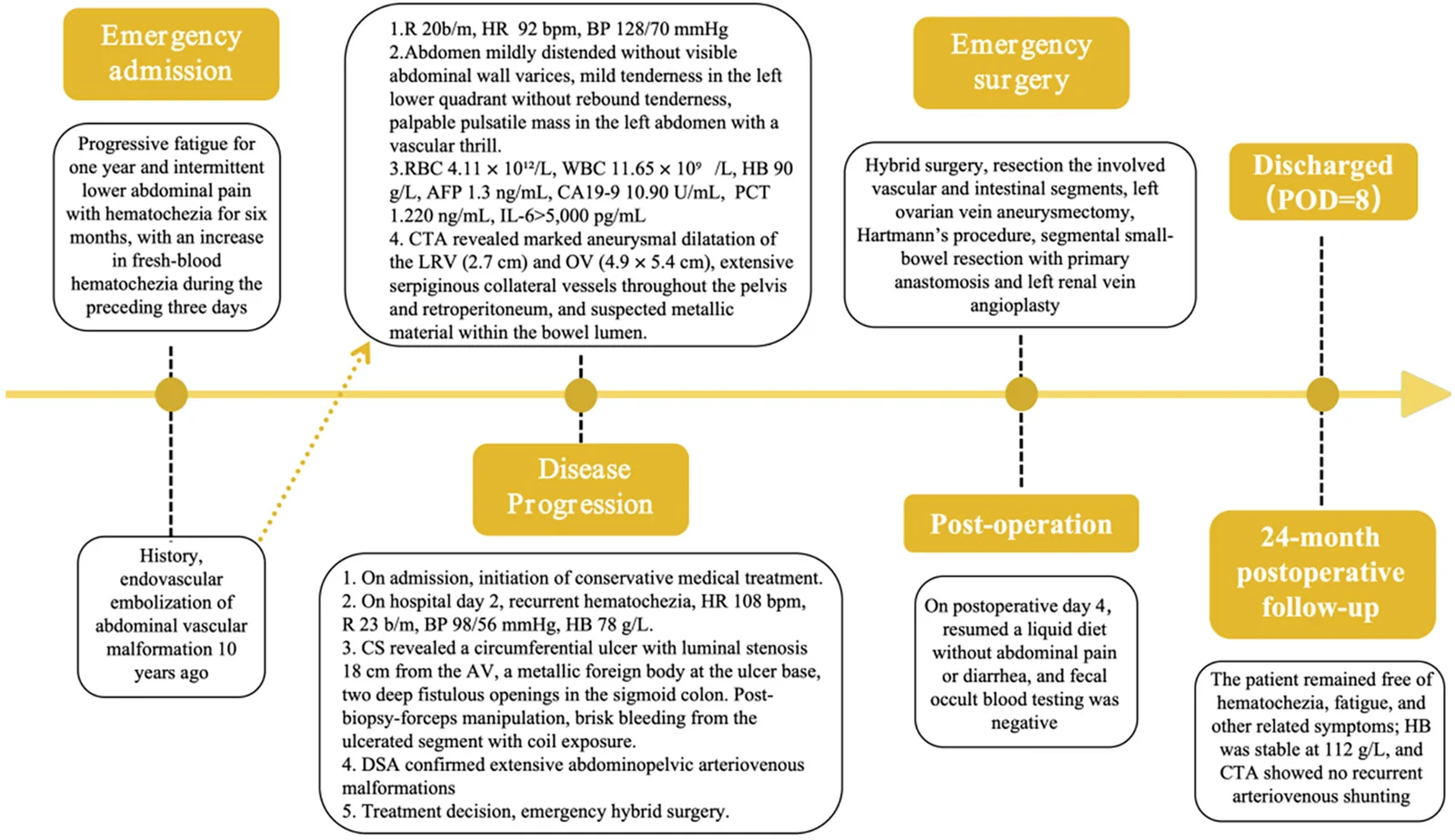
Clinical timeline of the patient's diagnosis, treatment, and follow-up.

Given the established diagnosis, extensive vascular and bowel involvement demonstrated by imaging, and ongoing active bleeding, together with the inability of supportive or endoscopic treatment alone to address the extensive high-flow vascular lesion and damaged bowel, the patient underwent emergency hybrid surgery. Laparotomy revealed multiple diffuse and tortuous abnormal vessels throughout the left abdomen, pelvis, and retroperitoneum, accompanied by tense aneurysmal dilatation of the left renal and ovarian veins. The vascular malformation was closely associated with the descending and sigmoid colon, and the left ureter was also surrounded by abnormal vessels. Intraoperative digital subtraction angiography (DSA) was first performed to delineate the vascular anatomy and guide vascular control. DSA demonstrated a complex and extensive high-flow inferior mesenteric arteriovenous malformation (IMAVM), characterized by a diffuse nidus composed of numerous tortuous abnormal vessels involving the sigmoid mesocolon and adjacent pelvic and retroperitoneal regions. The lesion was predominantly supplied by branches of the inferior mesenteric artery, with early venous drainage through the markedly dilated left ovarian and renal veins into the inferior vena cava (Figures 4A–D); recanalization of previously coil-embolized vessels was also observed (Figures 4A,B). The left renal vein, inferior mesenteric artery, and left common, internal, and external iliac arteries were then sequentially exposed. The inferior mesenteric and left internal iliac arteries were doubly ligated, resulting in a marked reduction in pressure within the abdominopelvic vascular malformation and venous aneurysms. The rectum and sigmoid colon were mobilized, the adherent ileum was carefully separated, and the abnormal vessels were ligated or oversewn while the left ureter was identified and protected. Then, we resected the abnormal vascular network constituting the arteriovenous malformation (AVM) (Figure 5A) and the involved colorectal segment (Figures 5C,D). We subsequently performed Hartmann’s procedure, resected the involved small-bowel segment, and completed a side-to-side anastomosis. The left ovarian venous aneurysm was excised (Figure 5D), and the aneurysmal segment of the left renal vein was resected, followed by venoplasty to preserve renal venous drainage (Figure 5B). Finally, bleeding points around the left ureter and on the surface of the abdominal aorta were repaired. During the procedure, 2 units of packed red blood cells and 200 mL of plasma were transfused to maintain hemodynamic stability. The operation was completed successfully without further complications. On postoperative day 4, the patient resumed a liquid diet without abdominal pain or diarrhea, and fecal occult blood testing was negative. Her postoperative course was uneventful, and she was discharged on postoperative day 8. Intestinal continuity was restored 6 months after the initial operation. At the 2-year follow-up, she remained free of hematochezia, fatigue, and other related symptoms; her hemoglobin level was stable at 112 g/L, and computed tomography angiography showed no recurrent arteriovenous shunting (Figure 5E).

**Figure 4:**
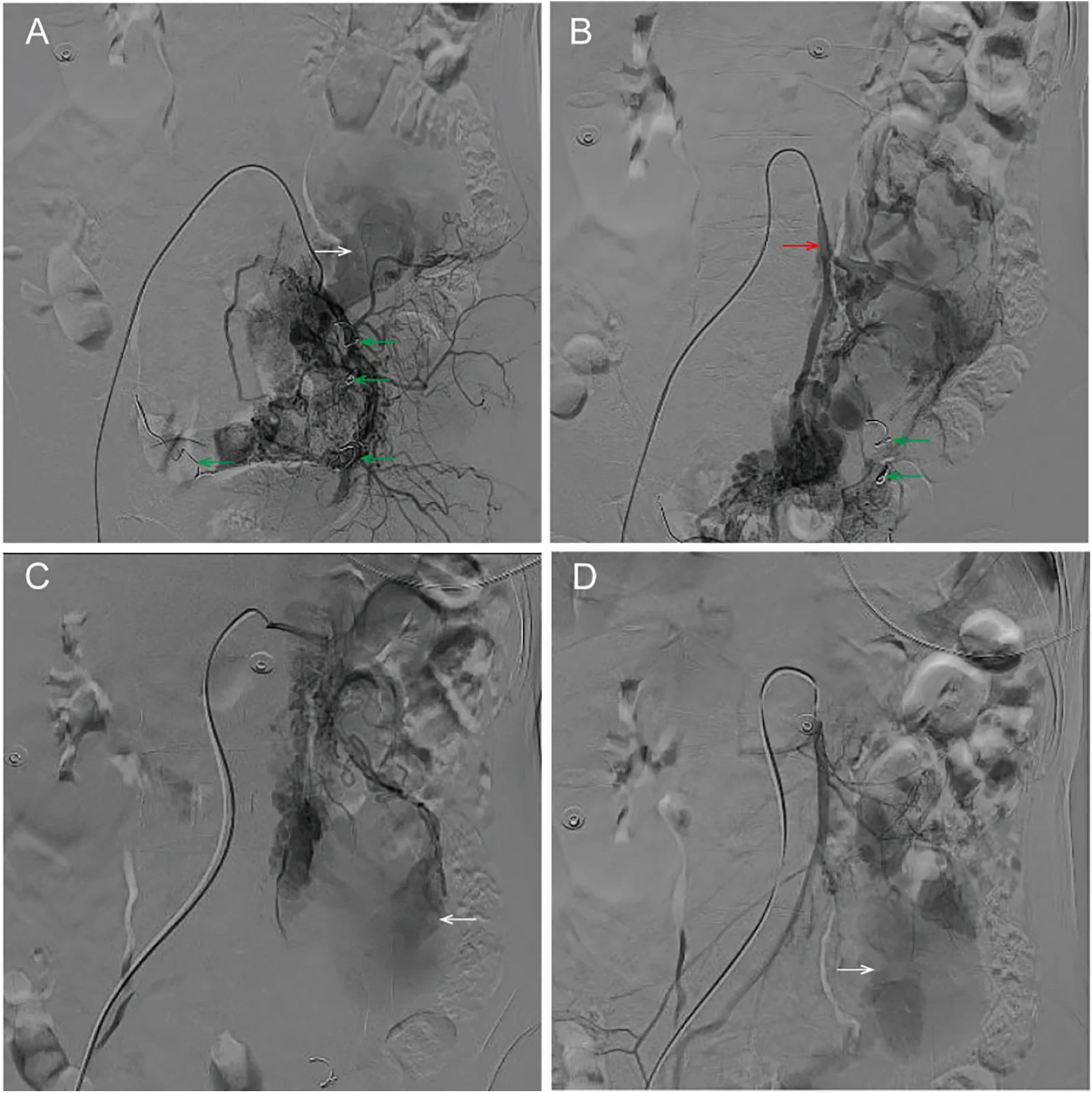
Intraoperative digital subtraction angiography findings. **(A,B)** Digital subtraction angiography demonstrated an inferior mesenteric arteriovenous malformation (red arrow), numerous abnormal vessels, early opacification of the left ovarian vein (white arrow), and recanalization of the previously embolized vessels (green arrows). **(C,D)** Angiographic images showed early opacification and aneurysmal dilatation of the left ovarian vein.

**Figure 5:**
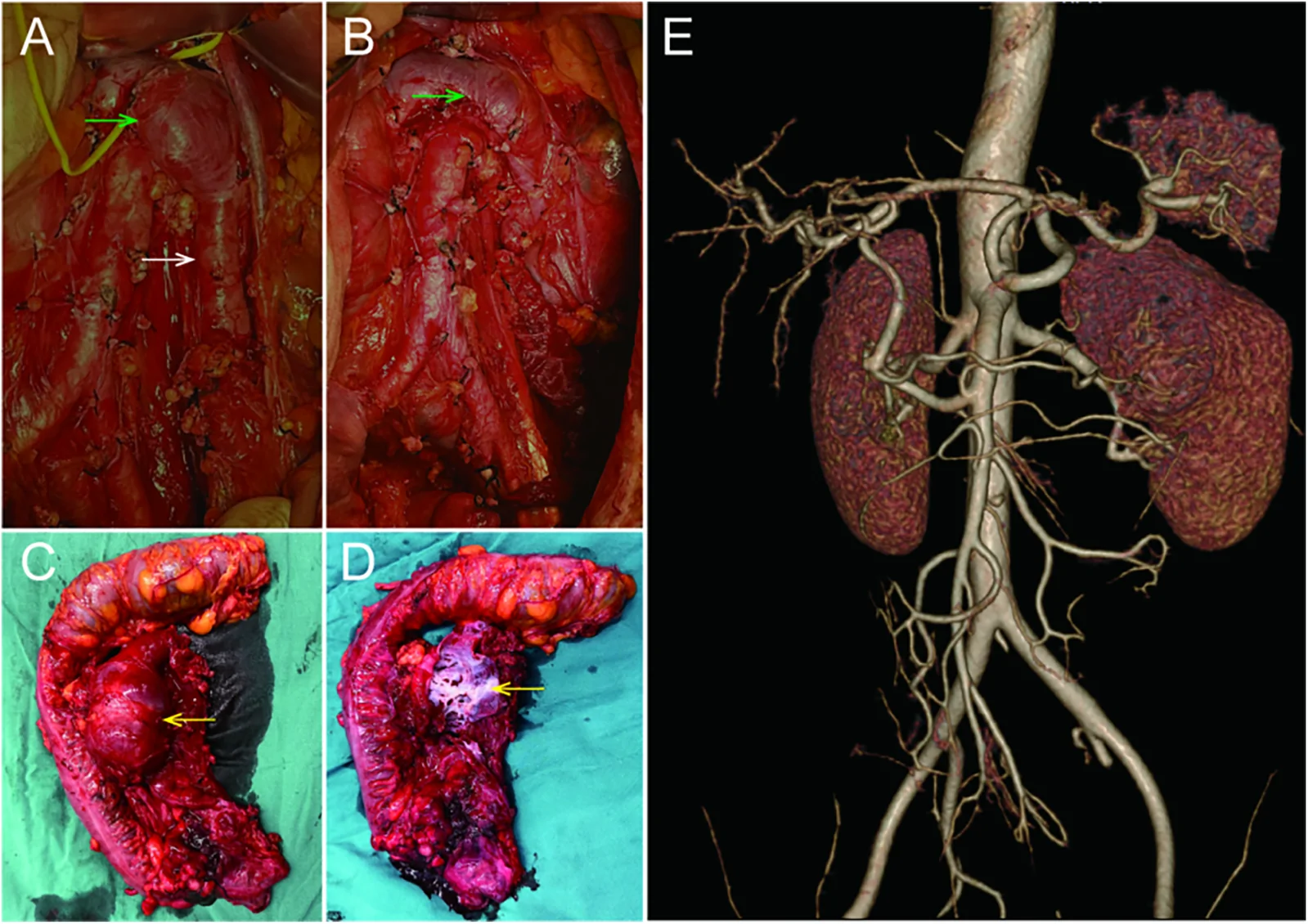
Operative findings and postoperative imaging. **(A)** Extensive dilated and tortuous vessels involving the mesentery and retroperitoneum before resection; the green and white arrows indicate the left renal vein and left ovarian vein, respectively. **(B)** Operative field after resection of the involved vascular lesions, showing angioplasty of the left renal vein (green arrow). **(C,D)** Gross and opened views of the resected sigmoid colon and mesentery demonstrate extensive vascular involvement and a conglomeration of abnormal vessels corresponding to the AVM nidus (yellow arrows). **(E)** Three-dimensional computed tomography angiography at the 2-year follow-up showing no recurrent arteriovenous shunting.

## Discussion

Endovascular embolization has become an important treatment option for symptomatic visceral arteriovenous fistulas and vascular malformations because it can rapidly reduce abnormal shunting with less procedural trauma than open surgery (4, 5). Coils are particularly suitable when controlled deployment and focal mechanical occlusion are required. Nevertheless, incomplete occlusion, persistent high-flow channels, recanalization, erosion, and migration remain clinically important concerns, especially when the embolization material is located adjacent to the gastrointestinal tract (6–10). Available reports indicate that coil-related complications may occur from several days to many years after embolization. Most reported cases have involved migration or erosion into the stomach or duodenum following embolization of the gastroduodenal, hepatic, splenic, or other visceral arteries (6–8), resulting in gastrointestinal injury and associated vascular complications. Colonic involvement, particularly migration into the sigmoid colon, appears to be considerably less common (9, 10). These delayed complications may emerge several years after the initial intervention and pose substantial diagnostic and therapeutic challenges.

Several mechanisms may contribute to the delayed development of bowel injury associated with coil migration. Persistent arteriovenous shunting may promote progressive enlargement of the feeding arteries and draining veins, thereby altering local shear stress and the interaction between the implanted material and adjacent tissues (1, 4, 11). Chronic arterial steal and venous hypertension may also contribute to ischemic-type bowel injury, edema, ulceration, and increased bowel-wall fragility (2, 3, 12–14). Against this background, a coil may gradually become unstable or mechanically erode an already compromised bowel wall. Conversely, ulceration and fistula formation may precede and subsequently expose a previously stable coil. Thus, delayed coil migration and chronic erosion should be regarded as complementary rather than mutually exclusive mechanisms.

For arteriovenous malformations, embolization strategies should be individualized according to lesion anatomy, hemodynamics, vascular access, proximity to vulnerable organs, and the need to preserve critical arterial inflow and venous drainage (4, 5, 11, 15). Detachable coils provide precise and controllable mechanical occlusion; however, they may be insufficient as the sole embolic agent for diffuse high-flow lesions and remain susceptible to compaction, recanalization, erosion, and migration. Vascular plugs are effective for occluding large, relatively straight vessels but require an adequate landing zone and a larger delivery system. Particulate agents can achieve distal devascularization, although their distribution is flow dependent and carries risks of non-target embolization and tissue ischemia. Liquid embolic agents provide deeper penetration into complex vascular networks. N-butyl cyanoacrylate produces rapid and permanent occlusion but can be difficult to control, whereas ethylene-vinyl alcohol copolymer permits slower and more controlled delivery at the expense of longer procedural and fluoroscopy times and the need for specialized equipment. Ethanol provides durable endothelial ablation but has a narrow therapeutic margin because of its limited controllability and substantial local and systemic toxicity (11, 15). Accordingly, complex high-flow lesions often require a tailored combination of embolic agents rather than reliance on a single material. In the present case, the records and angiographic images from the initial embolization were unavailable, preventing assessment of the original embolization strategy, the extent of shunt exclusion, and the initial coil position. Nevertheless, delayed coil migration into the sigmoid colon 10 years after embolization underscores the potential for material-related complications to occur long after the initial procedure. Patients undergoing embolization of complex fast-flow vascular lesions should therefore receive long-term clinical and imaging surveillance to detect residual or recurrent shunting, progressive vascular remodeling, venous dilatation, bowel ischemia, and delayed embolic material–related complications. The surveillance interval and duration should be individualized according to lesion anatomy, embolic material, residual flow, patient age, symptoms, and the feasibility of further intervention. Although detailed information regarding the patient's previous endovascular intervention could not be obtained, this rare case still provides clinically meaningful insights into diagnosis and management, suggesting that delayed embolization-related complications should be considered in patients with a history of endovascular treatment who present with recurrent gastrointestinal bleeding. It also illustrates the value of developing an individualized surgical strategy for complex vascular and bowel involvement.

In conclusion, delayed coil migration or erosion into the gastrointestinal tract is rare but potentially life-threatening. When exposed embolic material is accompanied by active bleeding, fistula formation, extensive vascular involvement, and structural bowel injury, surgery provides a direct and definitive treatment. Management should be individualized according to vascular anatomy, bowel involvement, embolic material, and the feasibility of endovascular, surgical, or hybrid reconstruction.

## Patient perspective

The patient reported resolution of gastrointestinal bleeding and fatigue after surgery and expressed satisfaction with the clinical outcome. She acknowledged the importance of long-term follow-up after vascular intervention.

## Data Availability

The original contributions presented in the study are included in the article/Supplementary Material, further inquiries can be directed to the corresponding authors.

## References

[1] Wassef M Borsik M Cerceau P Faucon B Laurian C Le Clerc N. Classification of vascular tumours and vascular malformations. Contribution of the ISSVA 2014/2018 classification. Ann Pathol. (2021) 41(1):58–70. 10.1016/j.annpat.2020.11.004 33309330

[2] Athanasiou A Michalinos A Alexandrou A Georgopoulos S Felekouras E. Inferior mesenteric arteriovenous fistula: case report and world-literature review. World J Gastroenterol. (2014) 20(25):8298–303. 10.3748/wjg.v20.i25.8298 25009407 PMC4081707

[3] Cubisino A Schembri V Guiu B. Inferior mesenteric arteriovenous fistula with colonic ischemia: a case report and review of the literature. Clin J Gastroenterol. (2021) 14(4):1131–5. 10.1007/s12328-021-01411-9 33837935

[4] Soulez G Gilbert P Giroux M-F Racicot J-N Dubois J. Interventional management of arteriovenous malformations. Tech Vasc Interv Radiol. (2019) 22(4):100633. 10.1016/j.tvir.2019.100633 31864530

[5] Golzarian J Sapoval MR Kundu S Hunter DW Brountzos EN Geschwind J-FH. Guidelines for peripheral and visceral vascular embolization training: joint writing groups of SIR, CIRSE, and CIRA. J Vasc Interv Radiol. (2010) 21(4):436–41. 10.1016/j.jvir.2010.01.006 20346881

[6] Palagiri AN Hamo F Samo S Chawla S. Coil erosion into the duodenum following arterial embolization. ACG Case Rep J. (2019) 6(8):e00195. 10.14309/crj.0000000000000195 31737725 PMC6791628

[7] Matsubara Y Lim LA Hijikata Y Hirata Y Yotsuyanagi H. Embolization coil migration in the stomach and spontaneous excretion: a case report and review of the literature. Radiol Case Rep. (2020) 15(7):1018–22. 10.1016/j.radcr.2020.04.053 32547670 PMC7283936

[8] Choi C-J Lim H Kim D-S Jeong Y-S Park S-Y Kim J-E. Massive duodenal bleeding after the migration of endovascular coils into the small bowel. Clin Endosc. (2019) 52(6):612–5. 10.5946/ce.2019.020 31104455 PMC6900307

[9] Doroodchi A Shukla M Ramirez M Agarwal G. Hypogastric artery–colonic fistula after coil embolization of left hypogastric artery aneurysm. Ann Vasc Surg. (2021) 76:599.e11–e14. 10.1016/j.avsg.2020.12.010 33508449

[10] Sexton JJ Whittaker DR. A noninterventional management approach to endovascular coil migration into the sigmoid colon. J Vasc Surg. (2015) 61(2):507–9. 10.1016/j.jvs.2013.09.033 24239522

[11] Yakes WF Rossi P Odink H. How I do it. Arteriovenous malformation management. Cardiovasc Intervent Radiol. (1996) 19(2):65–71. 10.1007/BF02563895 8729181

[12] Brandt LJ Feuerstadt P Longstreth GF Boley SJ. ACG clinical guideline: epidemiology, risk factors, patterns of presentation, diagnosis, and management of colon ischemia. Am J Gastroenterol. (2015) 110(1):18–44. 10.1038/ajg.2014.395 25559486

[13] Capron J-P Gineston J-L Remond A Lallement P-Y Delamarre J Revert R. Inferior mesenteric arteriovenous fistula associated with portal hypertension and acute ischemic colitis: successful occlusion by intraarterial embolization with steel coils. Gastroenterology. (1984) 86:351–5. 10.1016/0016-5085(84)90422-0 6690362

[14] Metcalf DR Nivatvongs S Andrews JC. Ischemic colitis: an unusual case of inferior mesenteric arteriovenous fistula causing venous hypertension. Dis Colon Rectum. (2008) 51(9):1422–4. 10.1007/s10350-008-9377-2 18521673

[15] Leyon JJ Littlehales T Rangarajan B Hoey ET Ganeshan A. Endovascular embolization: review of currently available embolization agents. Curr Probl Diagn Radiol. (2014) 43(1):35–53. 10.1067/j.cpradiol.2013.10.003 24290201

